# 5-α Reductase Inhibitors and Prostate Cancer Mortality

**DOI:** 10.1001/jamanetworkopen.2024.30223

**Published:** 2024-08-27

**Authors:** Robert J. Hamilton, Julian Chavarriaga, Najia Khurram, Cindy Lau, Jin Luo, Ning Liu, Maria Komisarenko, Girish Kulkarni, Christopher Wallis, David N. Juurlink, Neil Fleshner, Antonio Finelli

**Affiliations:** 1 Department of Surgical Oncology, Princess Margaret Cancer Centre, Toronto, Ontario, Canada; 2 Division of Urology, Department of Surgery, Princess Margaret Cancer Centre, Toronto, Ontario, Canada; 3 Institute for Clinical Evaluative Sciences, Toronto, Ontario, Canada

## Abstract

**Importance:**

5-alpha-reductase-inhibitors (5-ARIs) are approved for treating benign prostatic hyperplasia (BPH) and have been found to reduce prostate cancer (PCa) risk by 25%. However, trials also have shown 5-ARIs to be associated with high-grade PCa. Whether 5-ARIs increase mortality among those with a diagnosis of PCa remains unclear.

**Objective:**

To determine long-term outcomes of clinically localized PCa arising in individuals taking 5-ARIs compared with nonusers.

**Design, Setting, and Participants:**

This population-based cohort study was conducted between January 2003 and October 2017. Eligible participants were men aged 65 years or older in Ontario, Canada, who developed clinically localized PCa with complete pathological abstraction from the Ontario Health Administrative Databases. Data analysis occurred from November 2017 to November 2022.

**Exposure:**

5-ARIs before PCa diagnosis.

**Main Outcomes and Measures:**

The primary outcomes were overall mortality and PCa-specific mortality. Cause-specific hazard models with inverse probability treatment weights (IPTW) were used to examine associations of 5-ARI use with mortality outcomes. Sensitivity analyses based on prediagnostic 5-ARI use, Gleason score, comorbidity, 5-ARI indication, prostate-specific antigen modeling, and statin use were also performed.

**Results:**

The cohort included 19 938 patients with PCa. Of these, 2112 (10.6%; median [IQR] age, 74 [70-79] years) were 5-ARI users and 17 826 (89.4%; median [IQR] age, 71 [68-76] years) were nonusers. During a median (IQR) follow-up of 8.96 (6.28-12.17) years, 6053 (30.4%) died, including 1047 (5.3%) from PCa. 5-ARI use appeared to be associated with increased overall and PCa specific mortality in crude analyses; however, after IPTW, 5-ARI use was not associated with overall mortality (hazard ratio, 0.98; 95% CI, 0.90-1.07; *P* = .77) or PCa-specific mortality (hazard ratio, 1.02; 95% CI, 0.83-1.25; *P* = .84).

**Conclusions and Relevance:**

In this population-based cohort study of 5-ARI use prior to PCa diagnosis including long-term follow-up and clinicopathologic details, prediagnostic 5-ARI use was not associated with PCa-specific or all-cause mortality. This study offers reassuring safety data for patients using 5-ARIs before PCa diagnosis for both BPH and chemopreventive reasons.

## Key Points

**Question:**

Is 5-alpha-reductase-inhibitor (5-ARI) use prior to prostate cancer (PCa) diagnosis associated with overall mortality or PCa-specific mortality?

**Findings:**

In this cohort study of 19 938 patients with PCa who had long-term follow-up and detailed pathological information, prediagnostic 5-ARI use was not associated with overall mortality or PCa mortality.

**Meaning:**

These findings suggest that 5-ARIs are safe to use for benign prostatic hyperplasia and PCa chemoprevention.

## Teaser

This cohort study examines whether prediagnostic use of 5-alpha-reductase-inhibitors is associated with mortality outcomes among individuals with prostate cancer.

## Introduction

Approximately 1 in 8 men will receive a diagnosis of prostate cancer (PCa),^1^ which remains the second leading cause of cancer-related death in men in the US.^2^ Preventing PCa would dramatically reduce the physical and emotional burden faced by patients and families and the financial burden borne by the health care system.^3^ However, despite decades of research, no medications have been approved for PCa prevention.

The 5-α reductase inhibitors (5-ARIs) finasteride and dutasteride are the most promising candidates to date, but also the most controversial.^3,4,5,6^ The 5-AR enzyme converts testosterone into dihydrotestosterone (DHT), the most bioactive androgen in prostatic tissue. It is responsible for the embryologic development and growth of the prostate^7^ and the promotion of PCa.^8^ Tissue studies show that 5-ARIs produce dramatic decreases in intraprostatic DHT concentrations,^9,10^ identical to those observed after androgen deprivation therapy (ADT).^11^

In a randomized clinical trial of more than 18 000 men,^6^ finasteride was shown to reduce PCa risk by 25%. In a randomized clinical trial of 6729 men,^4^ dutasteride conferred a 23% reduction in PCa reduction. These risk reductions translate into a number needed to treat to prevent 1 case of PCa of approximately 20.^4,5,6^ If the story ended there, many individuals worldwide would undoubtedly be taking a 5-ARI for PCa chemoprevention. However, these 2 trials^4,6^ have been criticized because of the increased risk of high-grade tumors observed among those who used 5-ARIs. In the Thompson et al trial,^6^ the proportion of high-grade tumors (Gleason score ≥7) was 27% higher in the finasteride group, and in the trial by Andriole et al^4^ and Musquera et al,^12^ although no significant difference in tumors with a Gleason score of 7 or greater was reported, there was an observed increased risk in tumors with a Gleason score of 8 or greater, but this finding was not significant (29 participants [0.9%] vs 19 participants [0.6%]; *P* = .15).^4,12^

Not only is there concern that 5-ARIs may induce higher-grade tumors, but it is also unknown whether tumors arising in a setting of depleted DHT, regardless of grade, have more aggressive biological behavior compared with tumors arising in a normal hormonal milieu.^13,14,15,16^ The more important question is not what grade of cancer occurs despite 5-ARIs, but the long-term outcomes of cancers arising in patients taking 5-ARIs prior to PCa diagnosis. The aim of this study is to examine the association of 5-ARI use prior to PCa diagnosis with survival outcomes in a population-based cohort with long-term follow-up and detailed pathological information.

## Methods

### Data Sources

This cohort study was approved by the University Health Network institutional review board with a waiver of informed consent and followed the Strengthening the Reporting of Observational Studies in Epidemiology (STROBE) reporting guideline. We used the Ontario Cancer Registry (OCR), which captures 95% of newly diagnosed PCa in Ontario, Canada; the Ontario Health Insurance Plan database, which tracks claims paid to physicians (eg, surgery or radiotherapy); the Registered Persons Database (RPDB), which contains demographics and vital status; and the Ontario Drug Benefit (ODB) database which contains information on all outpatient prescriptions for patients aged 65 years or older. Between January 2003 and October 2017, we reviewed 128 017 patients with PCa, and identified a total of 65 626 patients aged 65 years or older who received a diagnosis of PCa and who were treated with radiation therapy, active surveillance, ADT, or radical prostatectomy. Of these patients, 46 337 had pathology reports available and variables of interest were manually abstracted by team members (N.K. and M.K.) at the Princess Margaret Cancer Centre. Participants who had data for variables of interest available (age, income quintile, rurality, stage, morphology type, laterality, prostate-specific antigen [PSA], total Gleason score, total number of positive cores, maximum percent core, primary treatment, and prior use of statins or metformin) were included in the analysis. Using the ODB, patients were classified as 5-ARI users based on any record of more than one 5-ARI prescription filled prior to their diagnosis. We used a 1-year look-back window to ensure we captured 5-ARI use. Survival outcomes and death date were obtained from the RPDB. Specific pathological details were manually abstracted from biopsy and prostatectomy pathology reports from the OCR.

Data were deidentified and available under a data use agreement with the OCR and Institute for Clinical Evaluative Sciences (ICES). These datasets were linked using unique encoded identifiers and analyzed at ICES.

### Case Definitions and Variables

PCa cases were identified by using *International Classification of Diseases for Oncology, Third Edition* (*ICD-O-3*) site code C61.9 and behavior code 3 (80003, 80103, and 81403). Age at diagnosis was grouped as 66 to 75 years, 76 to 84 years, and 85 years or older. Tumor stage was defined by the American Joint Committee on Cancer TNM staging system. Stage groups were categorized as I, II (A and B), III, IV, and V. Gleason score on needle core biopsy was categorized into 2 to 6, 7, 8, and 9 to 10. PSA at diagnosis was expressed in nanograms per milliliter (to convert to micrograms per liter, multiply by 1), and grouped into 0.0 to 6.0 ng/mL, 6.1 to 8.0 ng/mL, 8.1 to 15.0 ng/mL, and greater than 15.0 ng/mL. Neighborhood income quintile was obtained by linking postal codes from the RPDB to Statistics Canada Postal Code Conversion Files. For this measure we attributed median household income of each neighborhood (obtained from census data) to all persons living in that neighborhood. Neighborhoods were then ranked by quintile, from poorest (1) to wealthiest (5) within areas assigned by Statistics Canada. Primary treatment within 1 year of diagnosis was categorized into radical prostatectomy, radiation therapy, ADT, and active surveillance. Comorbidity scores were calculated using Johns Hopkins Adjusted Clinical Groups Case-Mix System, assigning a specific weight to each adjusted diagnostic group (low, ≤5; intermediate, 6-9; high, ≥10). Aggregated diagnosis groups were used to measure the number of chronic conditions per individual within each cohort and to identify individuals with the frailty marker.

### End Points

The primary end point was overall survival. The secondary end point was PCa-specific mortality, defined according to either clinical documentation or inclusion of PCa as a primary cause of death on the death certificate.

### Statistical Analysis

We used standardized differences for comparison of baseline clinical and demographic characteristics between 5-ARI users and nonusers. Inverse probability treatment weights (IPTWs) were calculated from a logistic regression model adjusting for prognostic covariates (age, income quintile, rurality, stage, morphology type, laterality, PSA, total Gleason score, total number of positive cores, maximum percent core, primary treatment, prior statin, posttreatment statin, prior metformin, and posttreatment metformin). We calculated stabilized IPTW by incorporating the marginal probability of receiving the 5-ARI in the weight calculation to adjust for extreme weights that can arise from extreme propensity scores. Stabilized IPTW were used in all weighted analyses. Kaplan-Meier curves and IPTW weighted curves were used to compare overall survival and PCa-specific mortality probabilities between the 2 study groups. IPTW Cox models were built to investigate the effect of 5-ARI use. Estimates are reported as hazard ratios (HRs) with their 95% CIs. We used cause-specific hazards models to generate the hazard ratio of cancer-specific death in the 5-ARI group compared with the nonuser group, taking death due to other causes as a competing event. Additionally, we used cumulative incidence function plots to visualize the association of 5-ARIs use with overall mortality and PCa-specific mortality. Furthermore, we calculated the cumulative incidence of death using attained age as a time scale among those who did and did not use 5-ARIs. For the time scale, we assumed the index date (date of PCa diagnosis) as time 0. We used attained age as a time scale and the cumulative incidence of death was calculated at each year of age instead of each year of follow-up, thus putting patients with the same age in the risk set together, allowing for a nonparametric adjustment for age effect.

We conducted sensitivity analyses repeating the primary analysis of 5-ARI use and association with overall survival and PCa-specific survival using the following 5 restrictions. First, to minimize a potential healthy-user effect, we limited the analysis to only those with a low comorbidity score (aggregated diagnosis group ≤5). Second, to create a more homogenous cohort in unmeasured characteristics, we used an active comparator approach and included only statin users as a marker of those who actively seek preventive care. Third, to minimize bias by indication, we limited the analysis to patients who had no history of benign prostatic hyperplasia (BPH) surgery or urinary retention to enrich the analysis with use of 5-ARI as a primary prevention. Fourth, to explore whether 5-ARI outcomes differ based on tumor differentiation and/or advanced stage, we ran separate models within strata of Gleason scores (sum ≤6, ≥7, or ≥8) and restricted to only those with locally advanced or advanced disease (cancer stage III or IV). Fifth, given that 5-ARIs reduce PSA values by a factor of 0.5 ng/mL, we entered the PSA covariate for the 5-ARI group by multiplying it by 2.

All analyses were completed with SAS statistical software version 9.4 (SAS Institute). *P* values were 2-tailed, and statistical significance was set at *P* = .05. Data analysis was conducted from November 2017 to November 2022.

## Results

**Figure 1.**
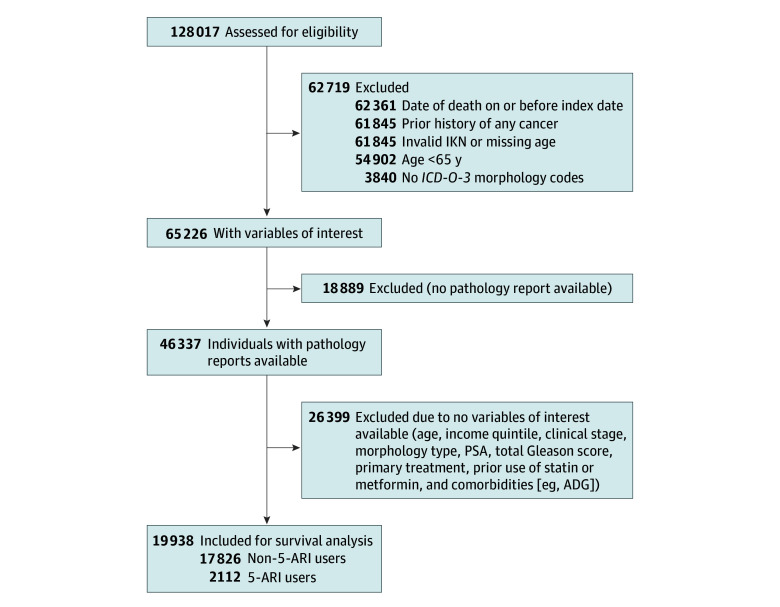
Study Flow Diagram 5-ARI indicates 5-α reductase inhibitor; ADG indicates aggregated diagnosis group; *ICD-O-3, International Classification of Diseases for Oncology (Third Edition);* IKN, Institute for Clinical Evaluative Sciences (ICES) key number; PSA, prostate-specific antigen.

We identified 128 017 individuals with PCa, of whom 46 337 had pathology reports available. After exclusions, including 26 399 missing a complete set of variables of interest, 19 938 formed our final cohort (Figure 1). Of the 19 938 patients, 2112 (10.6%; median [IQR] age, 74 [70-79] years) had received 5-ARIs prior to PCa diagnosis, while 17 826 (89.4%; median [IQR] age, 71 [68-76] years) had not. The Table summarizes the distribution of baseline characteristics between 5-ARI users and nonusers before and after IPTW. Some important differences included that 5-ARI users were older, had more comorbidities, and were more likely to have had ADT alone or active surveillance as their primary treatment. While they were more likely to have had a Gleason score of 8 or greater, they had a lower number of positive cores on average. After IPTW, no variable had a standardized difference greater than 0.10 (Table).

**Table.** Baseline Characteristics by 5-ARI Use Prior to Prostate Cancer Diagnosis

Variables	Participants, No. (%) (N = 19 938) [pre–inverse probability of treatment weighting]		Standardized difference	Participants, No. (%) (N = 2920.57) [post–inverse probability of treatment weighting]		Standardized difference
	No 5-ARI use (n =17 826)	5-ARI use (n =2112)		No 5-ARI use (n = 831.87)	5-ARI use (n =2088.70)	
Age at diagnosis, median (IQR), y	71 (68-76)	74 (70-79)	0.36	72 (68-76)	72 (68-76)	0.00
Neighborhood income, quintile						
1	2868 (16.1)	342 (16.2)	0.36	2870.35 (16.1)	329.06 (15.8)	0.00
2	3499 (19.6)	430 (20.4)	0.00	3516.59 (19.7)	428.96 (20.5)	0.02
3	3439 (19.3)	430 (20.4)	0.02	3459.54 (19.4)	391.24 (18.7)	0.02
4	3644 (20.4)	404 (19.1)	0.03	3620.59 (20.3)	418.78 (20.0)	0.01
5	4376 (24.5)	506 (24.0)	0.03	4364.80 (24.5)	520.66 (24.9)	0.01
Rural						
No	15 487 (86.9)	1878 (88.9)	0.01	15 530.70 (87.1)	1817.94 (87.0)	0.00
Yes	2339 (13.1)	234 (11.1)	0.06	2301.17 (12.9)	270.76 (13.0)	0.00
Stage						
I	1656 (9.3)	252 (11.9)	0.06	1706.45 (9.6)	203.74 (9.8)	0.01
IIA	2994 (16.8)	303 (14.3)	0.09	2949.33 (16.5)	343.52 (16.4)	0.00
IIIB	3049 (17.1)	465 (22)	0.07	3139.01 (17.6)	363.25 (17.4)	0.01
III	2421 (13.6)	437 (20.7)	0.12	2555.92 (14.3)	290.16 (13.9)	0.01
IV	1537 (8.6)	174 (8.2)	0.19	1531.81 (8.6)	186.37 (8.9)	0.01
Unknown	726 (4.1)	184 (8.7)	0.01	817.12 (4.6)	96.48 (4.6)	0.00
Aggregated diagnosis group, median (IQR)	7 (5-9)	8 (6-10)	0.40	7 (5-9)	7 (5-10)	0.01
Laterality						
Left	4079 (22.9)	574 (27.2)	0.00	4162.08 (23.3)	487.40 (23.3)	0.0
Right	4272 (24.0)	532 (25.2)	0.01	4298.27 (24.1)	512.00 (24.5)	0.01
Bilateral	9475 (53.2)	1006 (47.6)	0.01	9371.52 (52.6)	1089.30 (52.2)	0.01
Prostate specific antigen, ng/mL						
0.0-6.0	4646 (26.1)	615 (29.1)	0.24	4703.03 (26.4)	552.15 (26.4)	0.0
6.1-8.0	3901 (21.9)	401 (19.0)	0.24	3849.10 (21.6)	445.76 (21.3)	0.01
8.1-15.0	5573 (31.3)	583 (27.6)	NA	5505.32 (30.9)	638.74 (30.6)	0.01
>15.0	3706 (20.8)	513 (24.3)	0.10	3774.42 (21.2)	452.05 (21.6)	0.01
Gleason score						
1-6	6095 (34.2)	691 (32.7)	0.03	6066.02 (34.0)	703.51 (33.7)	0.01
7	8521 (47.8)	813 (38.5)	0.11	8343.36 (46.8)	970.96 (46.5)	0.01
8	1423 (8.0)	241 (11.4)	0.07	1490.45 (8.4)	182.71 (8.7)	0.01
9-10	1787 (10.0)	367 (17.4)	0.07	1932.05 (10.8)	231.52 (11.1)	0.01
Proportion of prostatic tissue involved by tumor for core, median (IQR)	35 (10-70)	30 (10-75)	0.08	35 (10-70)	35 (10-70)	0.03
Primary treatment						
Radical prostatectomy	3979 (22.3)	286 (13.5)	0.08	3813.27 (21.4)	443.52 (21.2)	0.00
Radiation	6632 (37.2)	695 (32.9)	0.03	6541.51 (36.7)	690.32 (33.0)	0.08
Androgen deprivation therapy	2857 (16.0)	478 (22.6)	0.19	2987.77 (16.8)	361.17 (17.3)	0.01
Active surveillance	4358 (24.4)	653 (30.9)	0.12	4489.32 (25.2)	593.70 (28.4)	0.07
Prior statin use	8377 (47.0)	1164 (55.1)	0.22	8533.11 (47.9)	992.11 (47.5)	0.01
Prior metformin use	2223 (12.5)	300 (14.2)	0.23	2257.01 (12.7)	267.76 (12.8)	0.01
Poststatin use	12 071 (67.7)	1457 (69.0)	0.09	12 095.31 (67.8)	1377.08 (65.9)	0.04
Postmetformin use	3853 (21.6)	470 (22.3)	0.17	3864.15 (21.7)	442.38 (21.2)	0.01
Positive cores, median (IQR)	4 (2-6)	3 (2-6)	0.15	4 (2-6)	3 (2-6)	0.15

Abbreviations: 5-ARI, 5-α reductase inhibitor; NA, not applicable.

Conversion factor: To convert prostate-specific antigen to micrograms per liter, multiply by 1.

### Overall Survival

**Figure 2.**
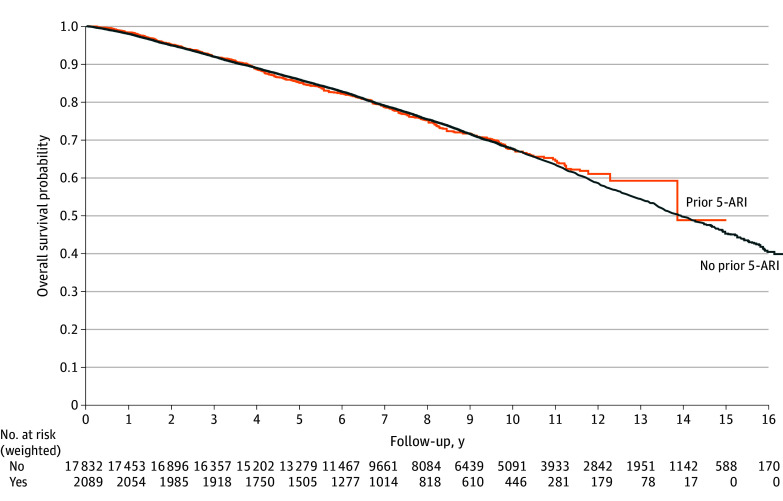
Overall Survival Comparing Those With and Without Prior 5-α Reductase Inhibitor (5-ARI) Use Kaplan-Meier curve with inverse probability of treatment weighting.

The median (IQR) follow-up for the cohort was 8.96 (6.28-12.17) years, during which 6053 patients (30.4%) died, including 1047 deaths (5.3%) from PCa. On crude analysis, 5-ARI use prior to PCa diagnosis was associated with worse overall survival (eFigure 1 in Supplement 1). However, after IPTW, 5-ARI use was not associated with overall survival on Kaplan-Meier estimates (log-rank *P* = .77) (Figure 2), or in our weighted Cox model (HR, 0.98; 95% CI, 0.90-1.07). In exploring the weight of influence of confounding variables, we observed the inclusion of age had the biggest effect size. To ensure our null observation was not inaccurate, we first explored within strata of age and observed no association of 5-ARI use with survival for patients aged 66 to 75 years (HR, 1.05; 95% CI, 0.92-1.20), 76 to 84 years (HR, 1.12; 95% CI, 0.99-1.26), and 85 years or older (HR, 1.01; 95% CI, 0.81-1.37) (eFigure 2 in Supplement 1). Finally, we used attained age as a time scale, and the cumulative incidence of death was calculated at each year of age instead of each year of follow-up, thus putting patients of same age in the risk set together, allowing for a nonparametric adjustment for age effect. In doing so, we again found no association of 5-ARI use with overall survival (HR, 1.02; 95% CI, 0.94-1.11; *P* = .54).

### PCa-Specific Mortality

**Figure 3.**
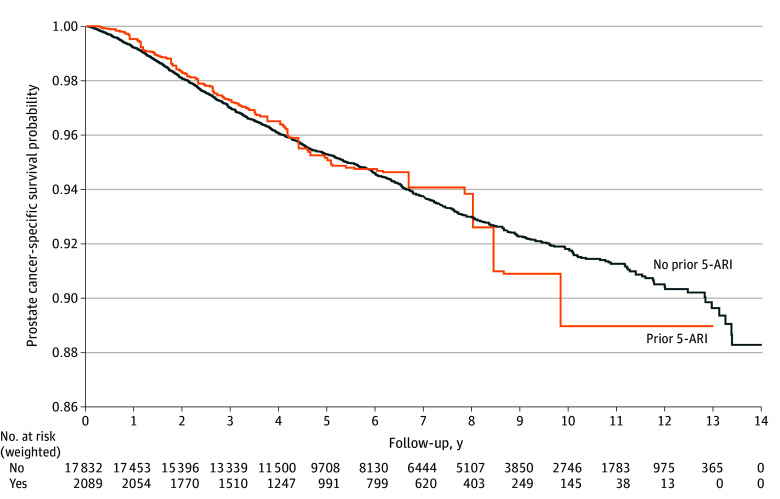
Prostate Cancer–Specific Mortality Comparing Those With and Without Prior 5-α Reductase Inhibitor (5-ARI) Use Kaplan-Meier curve with inverse probability of treatment weighting.

In unadjusted analyses, like overall survival, 5-ARI use prior to PCa diagnosis was associated with worse PCa-specific mortality. However, after IPTW, 5-ARI use was no longer associated with PCa-specific survival (HR, 1.02; 95% CI, 0.83-1.25; *P* = .84) (Figure 3).

### Sensitivity Analyses

**Figure 4.**
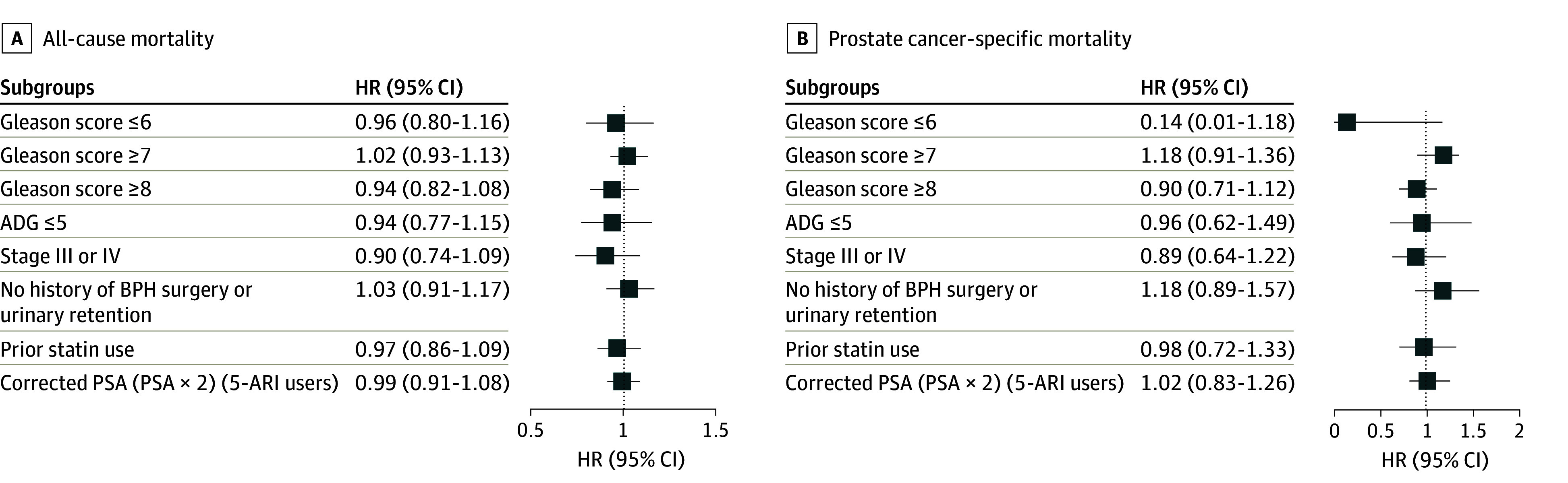
Subgroup Analysis Comparing 5-α Reductase Inhibitor (5-ARI) Use Prior to Prostate Cancer Diagnosis 5-ARI, 5-α reductase inhibitor; ADG indicates aggregated diagnosis group; BPH, benign prostatic hyperplasia; PSA, prostate-specific antigen.

We conducted a series of preplanned sensitivity analyses to ensure our null result was not a product of method of analysis (Figure 4). Overall, there continued to be no association of 5-ARI use with overall survival or PCa–specific survival. Of particular importance, given prior observations of 5-ARIs possibly inducing high-grade disease, when analyses were restricted to either low-grade tumors (Gleason score ≤6), or high-grade tumors (Gleason score ≥7), no association of 5-ARI use with overall mortality or PCa-specific mortality was observed. Finally, a sensitivity analysis was performed to model the scenario in which patients had at least 2 filled 5-ARI prescriptions (1811 participants). In the weighted analysis, 5-ARI with 2 or more filled prescriptions similarly showed no association with overall mortality (HR, 1.00; 95% CI, 0.92-1.12; *P* = .87) or PCa-specific mortality (HR, 0.96; 95% CI, 0.77-1.20; *P* = .73).

## Discussion

Data from prospective randomized trials has shown 5-ARIs reduce PCa risk by 25%. However, 5-ARIs appear to be associated with high-grade PCa and may alter the intraprostatic hormonal environment; therefore, in this cohort study, we sought to examine the long-term outcomes of clinically localized PCa arising in men taking 5-ARIs as compared with nonusers.

In our large, population-based cohort with long follow-up, 5-ARI use was associated with higher Gleason grade disease. However, after IPTW, 5-ARI use prior to PCa diagnosis was not associated with overall survival or PCa-specific survival; this observation held after multiple sensitivity analyses. We believe our findings support the safety of using 5-ARIs both for BPH and PCa prevention indications.

The initial enthusiasm around the positive results from the Thompson et al trial^6^ and the Andriole et al^4^ and Musquera et al^12^ trial were dampened by the observation that most of the cancers that 5-ARIs prevented were low grade, while there appeared to be an increased risk of high-grade disease. In fact, the US Food and Drug Administration Oncology Drugs Advisory Committee, after reviewing the data, issued a black box label recommending against the use of 5-ARIs for cancer prevention. The prospect of prescribing otherwise healthy men 5-ARIs with the pitfall of potentially inducing high-grade disease and increasing PCa mortality has prevented physicians from adopting their widespread use.^17^

There is some evidence of adverse effects of tumors arising in a setting of low testosterone.^13,14^ Hypogonadal androgen levels have been associated with higher-grade disease, higher rates of extracapsular extension, and seminal vesicle invasion in small studies, but after controlling for pathological stage and grade, low testosterone has not been shown to be independently associated with progression after surgery.^13,14,18,19^ On the other hand, Hsieh et al^16^ found that in the tissue of men with BPH treated with 5-ARIs and androgen-dependent PCa cell lines exposed to 5-ARIs, there was upregulated levels of AR in comparison with untreated tissue. Such AR upregulation is one of the cellular hallmarks observed in castration-resistant PCa.

While the associations of 5-ARI use with patient characteristics at the time of diagnosis has been well studied, later outcomes of patients taking 5-ARIs at the time of diagnosis has not been sufficiently studied. Prediagnostic 5-ARI use in men with low-risk PCa followed by active surveillance was found not to be harmful and actually decreased pathological or therapeutic progression in the Fleshner et al,^20^ and similar results were found in a single center retrospective study with a nearly 7-year follow-up.^21^ However, these data do not address the question of prediagnostic 5-ARI use.

The few studies assessing the association of 5-ARI before PCa diagnosis with overall and cancer-specific mortality have had mixed results and with limitations (eTable in Supplement 1). In a large, prospective cohort study of 4383 men, Vaselkiv et al^22^ noted 5-ARI use was not associated with cancer-specific survival (HR, 0.78; 95% CI, 0.48-1.27) or overall survival (HR, 0.88; 95% CI, 0.72-1.07). However, the study was underpowered because only 235 men were taking 5-ARIs. A large population-based study from the UK^23^ observed 5-ARI use prior to diagnosis was not associated with either PCa-specific mortality (HR, 0.90; 95% CI, 0.73-1.13) or overall mortality (HR, 0.92; 95% CI, 0.80-1.07). However, follow-up was limited (mean follow-up, 4.5 years).^23^ In a small population-based cohort study by Kjellman et al,^24^ finasteride users had an increased risk of receiving a diagnosis of nonlocalized disease, but this did not translate into worse PCa-specific survival. However, the study only included 199 men treated with finasteride with a median 2.7-year follow-up.^24^ A post hoc analysis^25^ of the Thompson et al trial^6^ found no association of 5-ARI use with overall survival (HR, 0.93; 95% CI, 0.78-1.12). However, survival data was only available for 802 finasteride users and PCa-specific mortality could not be ascertained.^25^ Two recently published large population-based cohort studies using data from 5816^26^ and 1377^27^ 5-ARI users from Sweden showed that 5-ARI use before PCa diagnosis was not associated with PCa-specific mortality (HR 0.50; 95% CI, 0.27-0.91^27^; HR, 1.10; 95% CI, 0.70-1.47^26^) or all-cause mortality (HR 0.96; 95% CI, 0.91-1.02^27^). They employed Cox proportional hazard models, adjusting for age, PSA levels, comorbidities, education, as well as curative treatment; however, they were unable to adjust for more detailed pathological information.^26,27^

The aforementioned studies found similar results to our overall findings, with our study strengthened by numbers, follow-up, and ability to control for more detailed clinical and pathological variables. It is encouraging that, while the aforementioned studies and our study found no statistical association with mortality, all the hazard ratios favor a protective association. One further study^28^ that is important to mention is a population-based cohort of 80 875 men in the Veterans Affairs database. Sarkar et al^28^ found a higher PCa-specific mortality (HR, 1.39; *P* < .001) and all-cause mortality (HR, 1.10; *P* < .001) in 5-ARI users prior to PCa diagnosis. They also observed prediagnostic 5-ARI use was associated with a Gleason score of 8 or greater (locally advanced or advanced disease).^28^ These findings are similar to our findings in unadjusted analyses. Although they adjusted for some population-available variables, they could not adjust for detailed pathological variables as we did, and this may explain why we observed no association after IPTW. It is important to note that it is likely our study and the aforementioned studies underrepresent the safety of 5-ARIs because these analyses do not account for the men who never received a diagnosis of PCa because they took a 5-ARI.

### Limitations and Strengths

Our study has potential limitations. We did not have central pathologic review of Gleason score; this may have resulted in misclassification of cases by grade. Our study was limited to those aged 65 years and older because the ODB only provides medication coverage to this subset, which limited our ability to perform a time-dependent covariate analysis given that younger patients in our cohort only had a limited look-back window. Furthermore, we cannot account for migration in and out of Ontario. Finally, we were unable to control for subsequent treatments after the development of metastatic disease or castration resistant PCa, which may have been differentially received by 5-ARI users and could have influenced survival.

Nevertheless, this study boasts several strengths. To our knowledge, our study is one of the largest, population-based cohorts with one of the longest-follow-up periods reporting PCa-specific mortality to date. Our detailed demographic, clinical and especially pathological data collection allows for covariate adjustment lacking in other studies.

## Conclusions

In our cohort study, which had, to our knowledge, the largest combined population-based size, follow-up, and detailed pathological information to date, we observed that 5-ARI use prior to PCa diagnosis was not associated with overall or PCa-specific mortality compared with those who did not use 5-ARIs. Our results support the safety of 5-ARIs as agents to treat BPH, prevent PCa, and support the removal of label warnings on these products.

## Notes

Supplement 1. **eFigure 1.** Unweighted Kaplan-Meier Curves Comparing 5-ARI Users with No Users **eFigure 2.** Cumulative Incidence of Overall Mortality Using Attained Age as a Time Scale in 5-ARI Users and No Users **eTable.** Summary of Studies Analyzing 5-ARI Use and Mortality (Online Only)

Supplement 2.
Data Sharing Statement
