# Supplementary material for: 5-α Reductase Inhibitors and Prostate Cancer Mortality
Source: JAMA Netw Open. 2024 Aug 27;7(8):e2430223. doi: 10.1001/jamanetworkopen.2024.30223 (PMC11350475; doi:10.1001/jamanetworkopen.2024.30223)
Supplement: Supplement 1. — eFigure 1. Unweighted Kaplan-Meier Curves Comparing 5-ARI Users with No Users eFigure 2. Cumulative Incidence of Overall Mortality Using Attained Age as a Time Scale in 5-ARI Users and No Users eTable. Summary of Studies Analyzing 5-ARI Use and Mortality (Online Only) [file jamanetwopen-e2430223-s001.pdf]

Supplemental Online Content

Hamilton RJ, Chavarriaga J, Khurram N, et al. 5-α Reductase inhibitors and prostate cancer mortality. *JAMA Netw. Open*. 2024;7(8):e2430223. doi:10.1001/jamanetworkopen.2024.30223

eFigure 1. Unweighted Kaplan-Meier Curves Comparing 5-ARI Users with No Users
eFigure 2. Cumulative Incidence of Overall Mortality Using Attained Age as a Time Scale in 5-ARI Users and No Users
eTable. Summary of Studies Analyzing 5-ARI Use and Mortality (Online Only)

This supplemental material has been provided by the authors to give readers additional information about their work.

© 2024 Hamilton RJ et al. *JAMA Network Open.*

eFigure 1. Unweighted Kaplan-Meier Curves Comparing 5-ARI Users with No Users
A. Overall Survival (OS) and B. Prostate Cancer-Specific Mortality

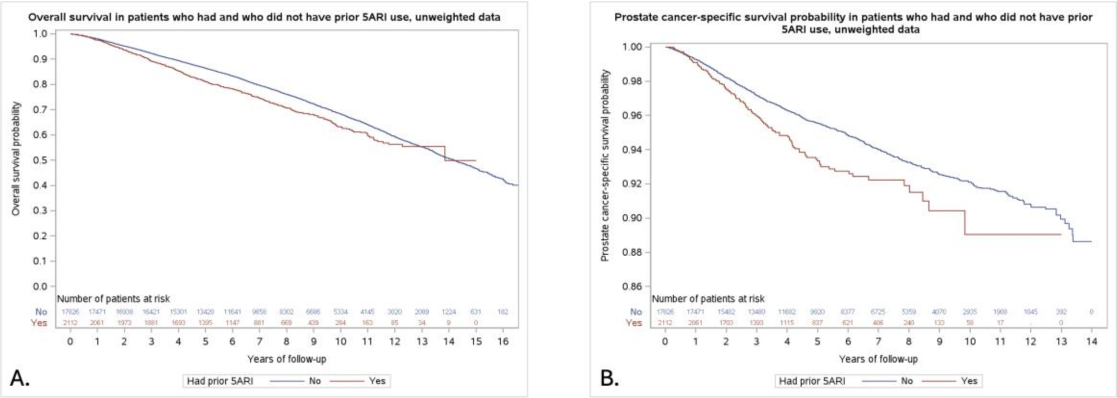

© 2024 Hamilton RJ et al. *JAMA Network Open.*

eFigure 2. Cumulative Incidence of Overall Mortality Using Attained Age as a Time Scale in 5-ARI Users and No Users

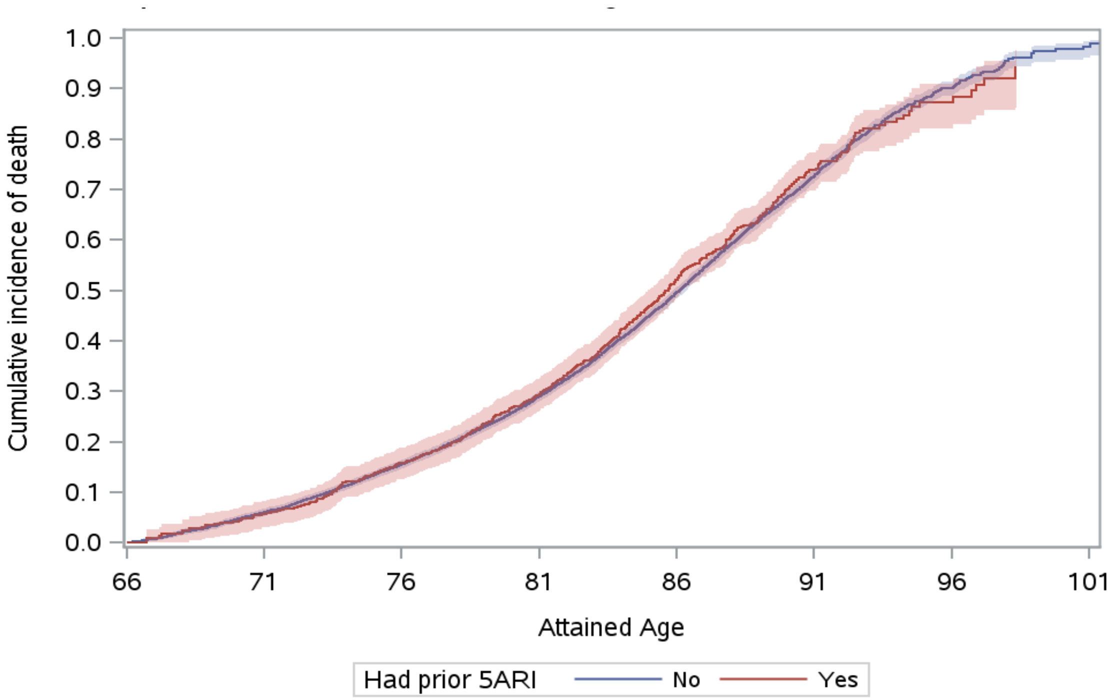

© 2024 Hamilton RJ et al. *JAMA Network Open.*

**eTable. Summary of Studies Analyzing 5-ARI Use and Mortality (Online Only)**							
**Authors**	**Year**	**Study Type**	**N (with PCa)**	**5-ARI users**	**Median follow-up**	**CSM**	**OM**
Kjellman et al	2013	Population-based	3,791	199	2.7 years	HR 0.94 (0.76–1.14)	HR 0.92 (0.77–1.10)
Thompson, Jr et al	2013	postHOC PCPT (RCT)	2401	989	18 years	0.93 (0.78–1.12)	(HR 0.93, 95% CI 0.78-1.12)
Azoulay et al	2015	Population-based	13,892	574	4.5 years	(HR 0.90, 95% CI 0.73-1.13)	(HR 0.92, 95% CI 0.80-1.07)
Sarkar et al	2019	Population-based (VA)	80,875	8,587	5.9 years	HR 1.39; 95% CI, 1.27-1.52	HR, 1.10; 95% CI, 1.05-1.15
Bonde Miranda et al	2020	Population-based (Sweden-PCBaSe)	89,227	5,816	5 years	1.10 (0.70-1.47)	N/A
Björnebo et al	2022	Population-based (Sweden)	16,181<br>(26,190)	1,377	8.2 years	0.50 (95% CI, 0.27-0.91).	0.96 (0.91-1.02)
Vaselkiv et al	2022	Cohort study	4,383	235	10.4 years	(HR 0.78, 95% CI 0.48-1.27)	HR 0.88, 95% CI 0.72-1.07)
**Hamilton et al**	2024	Population-based	19,938<br>(128,017 total)	2,112	8.96 years	HR 1.02 [95% CI 0.83-1.25]	HR 0.98 [95% CI 0.90-1.07

© 2024 Hamilton RJ et al. *JAMA Network Open.*
