# Supplementary material for: 5-α Reductase Inhibitors and Prostate Cancer Mortality
Source: JAMA Netw Open. 2024 Aug 27;7(8):e2430223. doi: 10.1001/jamanetworkopen.2024.30223 (PMC11350475; doi:10.1001/jamanetworkopen.2024.30223)
Supplement: Supplement 2. — Data Sharing Statement [file jamanetwopen-e2430223-s002.pdf]

Data Sharing Statement

Hamilton. 5-α Reductase Inhibitors and Prostate Cancer Mortality. *JAMA Netw Open*.
Published August 27, 2024. doi:10.1001/jamanetworkopen.2024.30223

Data
Data available: No
